# Gastric Symptoms in Spinal Disease

**Published:** 1903-01-31

**Authors:** 


### GASTRIC SYMPTOMS IN SPINAL DISEASE.

The mimicry of one disease by another and the difficulties thus thrown in the way of diagnosis are always matters of great interest to practitioners. Sir Frederick Treves[1] has recently pointed out how marked may be the mimicry of gastric troubles which arises in some cases of disease situated in the spine. A very short experience in the out-patient department of a large hospital, he says, will reveal a person or a child who is reputed to be suffering from some gastric trouble, but who is, in reality, the subject of caries of the spine. Such a child will complain of little more than an abiding pain in the stomach, the pain being probably about the umbilical region, in the area supplied by the eighth, ninth, and tenth dorsal nerves. The pain is actually due to the fact that there is an unsuspected caries of the dorsal spine, and that the nerves in question are pressed upon or are in some other way disturbed. Now and then such a condition is actually treated as a gastric trouble, and there is every excuse for a failure to recognise the *fons et origo mali* at a superficial inquiry. But the pain in these cases does not pass off; it survives the administration of rhubarb or castor oil or such aperient as may be in vogue; a little inquiry makes it evident that the pain is upon the surface. Moreover it abates when the patient lies down, the tongue is clean and there is no sickness. Examples of mimicry when they occur in adults are apt, however, to be even more deceptive, and this in spite of the fact that the adult has become more precise in the localisation of pain, and less apt to put everything down to stomach. In illustration of this Sir Frederick relates two cases. In the first the patient was a lady, about 45 years of age, who had entered a surgical home because it was considered probable that an abdominal operation would have to be performed on account of certain gastric symptoms. For several months she had complained of severe and distressing pain in the stomach which was said to be worse after food. There was vomiting which gave relief. The stomach was much dilated and the epigastric region was distinctly tender. The patient was quite an invalid, she had been under various forms of medical treatment and was no better, and she was now turning towards surgery as towards a forlorn hope. It was evident in this case that much of her trouble was due to chronic dyspepsia. Yet on further investigation it was found that there was the deformity of Pott's disease in the dorsal region, and that the patient had suffered from caries of the spine when a child. Although no complaint was made of the back it was probable that some recrudescence of the old bone trouble was at the root of the mischief. At any rate the pain, the sickness, and the dilatation of the stomach gradually vanished after no more elaborate treatment than a long confinement to bed in the recumbent position. In the second case the patient was a master-shipwright, aged 50, whose main symptom was pain after food. This increased and became intense, but it was always aggravated by food. During ten months he had been under constant medical care, and every method of treatment had been tried, yet he became steadily worse. He never once complained of his back, and he continued at his work in the shipbuilding yard, always maintaining that he was easier when at work. So anxious was he that something should be done for his relief and so gastric did his troubles seem, that in the end an exploratory operation was actually performed. The abdomen was opened but every organ was found to be normal. Several days after the operation the nurse drew attention to a slight lump between the patient's shoulders which she had discovered by accident. This proved to be a sarcoma growing from the mid-dorsal region of the spine. After a while paraplegia developed and he died. The moral to be drawn from these cases, says Sir Frederick, is this—that suspicion may be roused as to the genuineness of a gastric trouble when pain is the all-predominant symptom, when it is intense and persisting, and whem vomiting is at the same time either absent or insignificant.

[1] Practitioner, Jan.

